# Learning Soft Mask Based Feature Fusion with Channel and Spatial Attention for Robust Visual Object Tracking

**DOI:** 10.3390/s20144021

**Published:** 2020-07-20

**Authors:** Mustansar Fiaz, Arif Mahmood, Soon Ki Jung

**Affiliations:** 1 School of Computer Science and Engineering, Kyungpook National University, Daegu 41566, Korea; mustansar@knu.ac.kr; 2 Department of Computer Science, Information Technology University, Lahore 54000, Pakistan; arif.mahmood@itu.edu.pk

**Keywords:** Siamese networks, convolutional neural network, visual tracking, attentional mechanism

## Abstract

We propose to improve the visual object tracking by introducing a soft mask based low-level feature fusion technique. The proposed technique is further strengthened by integrating channel and spatial attention mechanisms. The proposed approach is integrated within a Siamese framework to demonstrate its effectiveness for visual object tracking. The proposed soft mask is used to give more importance to the target regions as compared to the other regions to enable effective target feature representation and to increase discriminative power. The low-level feature fusion improves the tracker robustness against distractors. The channel attention is used to identify more discriminative channels for better target representation. The spatial attention complements the soft mask based approach to better localize the target objects in challenging tracking scenarios. We evaluated our proposed approach over five publicly available benchmark datasets and performed extensive comparisons with 39 state-of-the-art tracking algorithms. The proposed tracker demonstrates excellent performance compared to the existing state-of-the-art trackers.

## 1. Introduction

Visual Object Tracking (VOT) is a promising, attractive, and challenging field in computer vision with a wide range of real-world applications including robotics [1], autonomous vehicles [2], video understanding [3], surveillance, and security [4]. Given the initial target location in the first frame of a video, the goal of an object tracker is to estimate the new target positions for subsequent frames. VOT is an active research area owing to the challenges such as occlusion, the presence of various types of noise, appearance and scale variations of the target, environmental changes, motion blur, illumination variations, and background clutter.

Correlation Filter-based Trackers (CFTs) have widely been used due to robustness and low computational complexity [5,6,7,8]. CFTs exploit circulant target structure to replace exhaustive spatial correlation operations with effective element-wise multiplication in the frequency domain to achieve high speed. Furthermore, CFTs use fairly simple image features such as a Histogram of Oriented Gradients (HOG), color-name [9] features, and a color-histogram. Despite these advantages, CFTs performance may drop under difficult and complex scenarios, due to these hand-crafted features. The performance of CFTs may be improved by using deep features, due to CFT’s inherent ability to learn discrimination between the target and the background.

Deep learning has widely been utilized in computer vision applications such as image classification [10,11], action recognition [12,13], semantic segmentation [14,15], and pose estimation [16,17]. Deep CFTs use deep features extracted from pre-trained networks over large benchmarks to compute discriminative features that result in improved performance compared to the hand-crafted CFTs [18]. Although many deep trackers [19,20,21,22,23,24,25] have demonstrated good performance, a limitation of pre-trained models is that they do not fully capture a specific target appearance. Therefore, many deep models [26,27,28,29] are trained end-to-end to further improve the performance. Another limitation is that during tracking, models update to adapt to new target appearances but may encounter an over-fitting problem. Moreover, online deep learning is computationally expensive and requires extra computational resources for feature extraction, update operations, and inference processes.

Recently, Siamese networks have been used for tracking to address some of these limitations. Siamese trackers are popular, owing to underlying properties such as competitive performance and computational efficiency. The basic principle behind a Siamese network is to find the similarity between input images. Siamese-based trackers can be simplified as a similarity learning problem: a similarity measure is learned in order to compute the similarity between the template image and the candidate image. Siamese trackers including [30,31,32,33,34] are computationally efficient but exhibit performance degradation under many scenarios. These trackers learn similarity by training offline on the large benchmarks and do not learn the most discriminative features for a specific target, which reduces the tracking performance. One approach is to assign different weights to the target parts on the basis of previous information [35,36], which is inaccurate. However, in the current manuscript, we introduce a soft-mask to highlight the target pixel information in the spatial domain during training. Our model estimates the new target information efficiently without computing the soft-mask during test time. Moreover, computation of the soft-mask for each frame requires extra computational cost, which reduces the tracking speed.

Recently, attention methods are studied to improve the feature representations in various computer vision applications [15,37,38,39,40]. Motivated by these applications, many trackers are utilizing different kinds of attentions in their tracking framework. For example, the tracker [41] is motivated by Selective Kernel Networks (SKNet) [39]. Authors computed separate feature weights for different convolutional layers. While tracker [42] is inspired by Convolutional Block Attention Module (CBAM) [37] and computes feature descriptors utilizing both global average and global maximum pooling layers for channel as well as spatial attention. The RASNet [43] model integrates three types of attentions such as residual attention, channel attention, and general attention to produce more discriminative target features. The authors of [15] utilized masks to modulate the features for video object segmentation.

We propose to integrate two different attention mechanisms in the Siamese tracking framework to emphasize discriminative channels and important spatial features in the latent space. The proposed attention mechanism boosts the underlying model to learn ’which’ and ’where’ target information should be highlighted. In this work, we emphasize to highlight target information in spatial as well as in latent space. We construct a soft-mask to highlight target pixel information in the spatial domain. However, in latent space, we integrate channel and spatial attention modules within the proposed tracking framework. Proposed channel attention computes two separate weight vectors and then fuses to highlight important features and reduces the less important features. Our proposed spatial attentional module computes weights for each feature map using a simple model without utilizing the global average and maximum pooling layers.

In particular, we propose a Soft-mask with Channel and Spatial attentional Siamese (SCS-Siam) tracking framework that learns both effective and discriminative features. We extend the underlying Siamese network architecture to exploit the target-specific information. The proposed SCS-Siam network encodes more target-specific information than contextual knowledge to learn the similarity between inputs and exhaustively exploits the template features to capture a specific target representation. A soft mask is computed to focus on the region of interest while suppressing background information in the image space. A spatial feature fusion operation is performed by combining the features from both the input image and the generated soft mask image. A channel attention mechanism is integrated for the template branch to produce robust discriminative target features by highlighting the most useful channels while suppressing the less important channels. A spatial attention mechanism is also utilized after channel attention to emphasize the important spatial regions within a channel for better target location identification. Both channel and spatial attentions are integrated within the Siamese framework using a skip connection. The proposed model is trained offline to enable generalized tracking and to enhance the robustness of the tracker. Extensive experiments are performed to evaluate the proposed SCS-Siam algorithm over five benchmark datasets including OTB2015 [44], TempleColor123 [45], UAV123 [46], VOT2016 [47], and VOT2017 [48].

The main contributions of the current manuscript are as follows:

- We propose a soft mask feature fusion mechanism to highlight the full target region compared to the background region. It helps the network to efficiently learn the target representation.
- A channel attentional mechanism is proposed to give the discriminative channels more importance.
- A spatial attention mechanism is proposed to emphasize the discriminative spatial locations within the target.
- Soft mask feature fusion with dual attention is integrated within a Siamese tracking framework using a skip connection to enhance the tracker ability to better discriminate target from the background.
- The proposed SCS-Siam tracker has shown excellent performance compared to 39 existing trackers over five benchmark datasets.

The rest of the paper is organized as follows. Section 2 presents the related work, Section 3 explains our proposed framework, Section 4 describes the experiments and evaluations and finally, Section 5 presents a conclusion and future research directions.

## 2. Related Work

In this section, we explore deep feature-based, Siamese-based, and attention-based trackers. Detailed research on trackers can be found in [18,49,50].

### 2.1. Deep Feature-Based Trackers

Deep learning has demonstrated ground-breaking performance in the tracking field. One notable limitation in visual tracking, however, is the limited availability of training samples. Most deep trackers use pretrained models to extract deep features trained over a large benchmark for object classification. Deep trackers [19,22] compute complementary features from shallow and semantic layers to obtain promising tracking results into the correlation filter. Moreover, deep features are exploited at various convolutional layers to boost the performance of visual trackers [20,21,24,51]. In contrast, a combination of features from different layers does not always guarantee a performance gain, mainly due to the increasing number of dimensions, assorted resolutions, and unknown target information [51]. VITAL [52] explored adversarial learning to produce efficient sample features and used a cost-sensitive loss function to leverage from the class imbalance. Other deep trackers [28,52,53,54,55] based on decision-making approaches have been proposed, such as Support Vector Machines (SVM), regression, and classification networks. Hong et al. [53] proposed CNN-SVM to perform a classification task using SVM and CNN models with saliency maps. MDNet [28] captured the domain-dependent information and performed tracking task as classification in a particle framework. Spatial and temporal information was encoded using CNNs for classification by Teng et al. [54]. Wang et al. [55] introduced a features-selection procedure based on a regression framework. These trackers use rich feature representations from deep networks but are limited in tracking performance and may drift, due to noisy updates during online learning. Moreover, additional computational cost is required to update these networks to capture a new target appearances.

### 2.2. Siamese Network-Based Trackers

Recently, Siamese networks have been utilized to develop robust visual trackers, drawing significant attraction in the visual tracking community owing to the real-time inference [30,33,34,56,57,58,59]. Siamese trackers learn the similarity between input images and cast the tracking problem as a matching problem. Siamese trackers perform tracking by comparing the initial target template features with search region features for every incoming frame. Siamese networks share the benefits of offline learning on large benchmarks to yield generic object tracking. Bertinetto et al. [30] developed SiameseFC computed complementary features using embedded CNN models and fused them to produce a response map. CFnet [34] was proposed to introduce a correlation layer in the template branch of the SiameseFC to produce superior results. GOTURN [33] was proposed to compute the similarity between two consecutive frames using a simple feed-forward network. Re3 [56] was proposed to utilize the recurrent network and obtain a better target representation. Guo et al. [57] proposed DSiam to suppress background information and performed online learning to capture target appearance variations. Tianyu and Antoni proposed MeemTrack [58] and integrated a dynamic memory network within a Siamese architecture. These methods are pre-trained on large benchmarks to learn similarity from pair-wise inputs; however, over-fitting may occur from learning on similar benchmarks. Moreover, these Siamese methods do not fully exploit the target information. The authors of [35,36] compute non-overlapping patches and assign weights to reflect the patch importance. In contrast, we extend the underlying Siamese architecture and compute a soft mask feature fusion to exploit the target information to highlight the objectness information while suppressing contextual information for better target feature representation.

### 2.3. Attention Mechanism-Based Trackers

An attention mechanism is popular across computer vision fields, including activity recognition [60], image classification [37,61], pose estimation [62], and semantic segmentation [40,63]. The RTT algorithm [64] uses multi-directional recurrent neural networks to produce saliency maps and draws attention to possible targets. Discriminative spatial attention is used by DAVT [65]. The SA-Siam algorithm [66] enhances the discriminative ability of the semantic branch by incorporating a channel attention module. The RASNet [43] model integrates three types of attentions: residual attention, channel attention, and general attention to produce more discriminative target features. Abdelpakey et al. [67] proposed DenseSiam and focused on non-local target features by using self attention in a template branch. The CSRDCF [68] algorithm constrains correlation filter learning by introducing spatial reliability that uses a spatial binary mask. In the current work, we focus on critical information and re-calibrate the channels for better discrimination. Proposed channel attention learns which deep channels should be highlighted for better target feature discrimination. We also exploit the target and background location information and focus where the pixel information should be highlighted or suppressed within each deep channel. The proposed channel and spatial attention modules exploit the intermediate features effectively to learn the ‘which’ and ‘where’ target information to focus or suppress.

## 3. Proposed SCS-Siam Network

**Figure 1:**
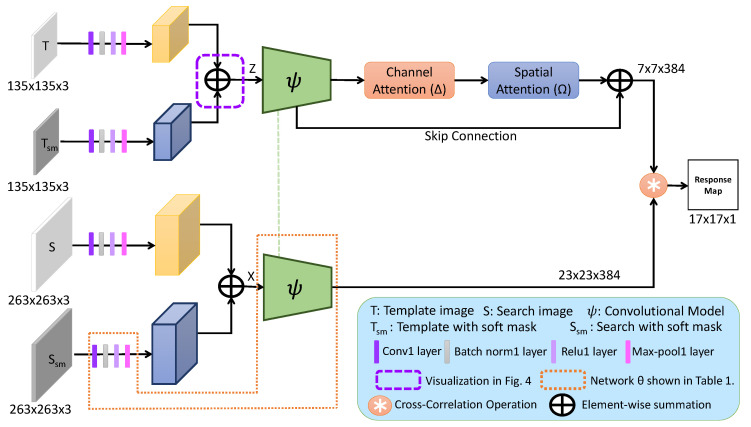
Framework of the proposed Soft-mask with Channel and Spatial attentional Siamese (SCS-Siam) tracker. The input images and the soft mask features are fused after the pooling layer for both exemplar and search branches to enhance target information. The fused features are forwarded to an embedded Convolutional Neural Network (CNN) model ψ to compute higher level discriminative features. Attention modules are integrated into the exemplar branch using a skip connection before the correlation operation. The output of channel attention is forwarded to the spatial attention module. Finally, a response map is produced using a cross-correlation operation between the exemplar branch features and search branch features.

The overall framework of the proposed Soft-mask with Channel and Spatial attentional Siamese (SCS-Siam) architecture is shown in Figure 1. Compared to the previous deep trackers, the proposed SCS-Siam learns the target object information by highlighting it while suppressing the contextual information by using a soft mask. As illustrated in Figure 1, the SCS-Siam fuses the soft mask features to adapt the learned target model with the appearance variations. Moreover, channel and spatial attention mechanisms are introduced to better utilize the intra-channel and inter-channel features for inference. The main components of the proposed SCS-Siam tracker are discussed in detail in the following sections.

### 3.1. Baseline SiameseFC Tracker

The SiameseFC works on the principle of template-matching and is a building block of our proposed tracker. The SiameseFC performs tracking by formulating the tracking problem as a general matching function. Embedded CNN models are used to compute the deep features for the input patches. SiameseFC is composed of two parallel branches known as the exemplar *z* branch and the search *x* branch. The goal of SiameseFC is to find the maximum similarity between the input images indicating the new target location. A response map g(z,x) is computed using a cross-correlation function as:

(1) g(z,x)=ψ(z)∗ψ(x)+b,

where ψ(·) represents the embedding model, ∗ represents the cross-correlation function, and *b* means the offset. The Equation (1) performs both feature representation and discriminative learning simultaneously, which may lead to the problem of over-fitting. However, the superior feature representation that preserves the target object information while reducing the impact of background can increase the tracking accuracy. In the current work, we present soft mask based feature fusion and two attention modules to improve the tracking performance.

### 3.2. Soft-Mask Feature Fusion

**Figure 2:**
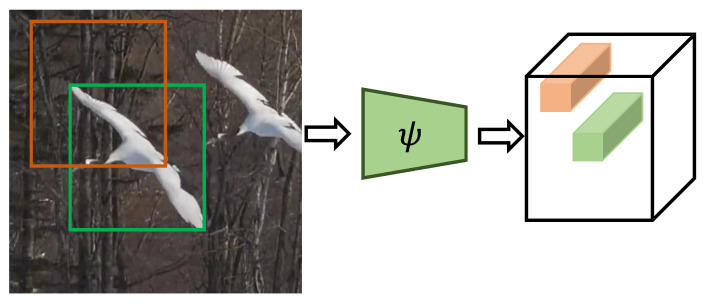
An example of a Siamese network that produces a feature space. Green and orange cubes in the cubic feature map indicate the features for green and orange regions in the image.

**Figure 3:**
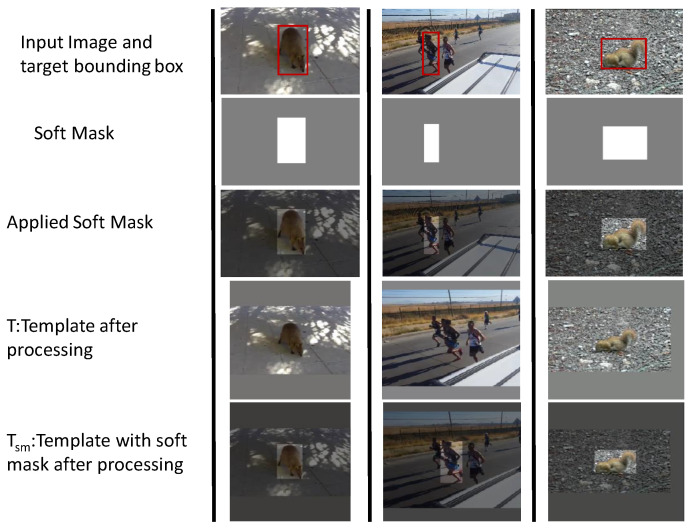
During training. Template with soft mask generated from three videos. The first row is the original input image, the red box shows the target position. The second row is the computed soft mask with σ= 0.5. The third row is the template with the soft-mask where the target bounding box has weight 1 while the background has weight 0.5. The fourth row is the template resized and padded with the image mean to bring the target object at the center position. The fifth row is the resized soft-mask again with the target object on the center and image mean values are padded to preserve the input size.

To develop a generic robust tracker and overcome the limitations in the baseline tracker, we propose a soft-mask feature fusion technique to suppress the background information compared to the target information. The underlying principle is that in Siamese networks, all components do not participate equally in the cross-correlation. For example, an object within the green bounding box should ponder more to compute a cross-correlation than the outside region of target bounding box, as illustrated in Figure 2. Moreover, to discriminate the target from the background, the visual difference between the target and the background must be more distinct. Thus, at the training stage, we introduced a soft mask generation process to train the network to exploit more target information for cross-correlation. The proposed mask generation module creates a contrast between the target and the background to enhance the discriminative ability of the tracker. The soft mask suppresses the background information outside the target bounding box. We constructed a masked image by providing lower weights σ ≤ 1 to the background. To do so, we multiply the input pixel values by a σ outside the bounding box. Figure 3 presents three examples of the soft mask generation.

Suppose, there is an input image *I* and its target bounding box BB in the first row of Figure 3. A soft-mask *m* is constructed on the basis of BB, as shown in the second row. The soft-mask is applied over the input image, as displayed in the third row. Template *T* and template with soft-mask $T_{sm}$ are generated after data curation. Similarly, Search image *S* and search with soft-mask $S_{sm}$ image are generated. Usually, the target can be on arbitrary location in the frame. To fit the model, we crop and resize the soft-masked image such that the target is centered (similar to [30]). Note that at the test time, the target region is not known precisely, therefore σ = 1 is used. During training, the value of σ = 0.90 is empirically found to be the best performer on OTB2015 and hence used for all datasets. The details of the empirical study are given in Table 9.

**Figure 4:**
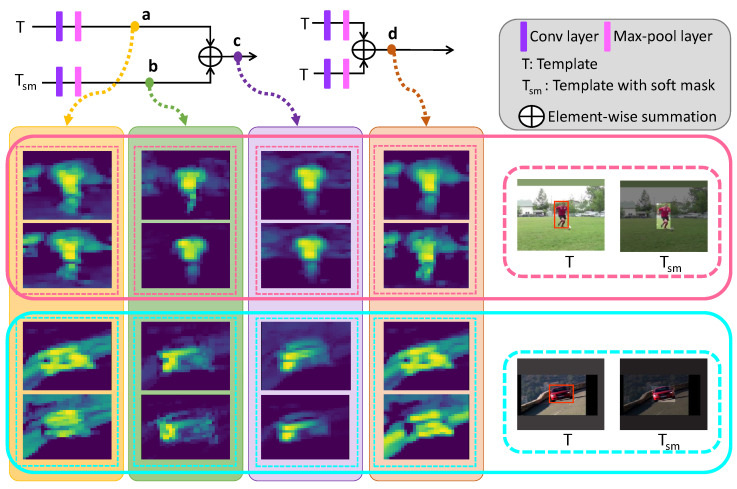
Applied soft-mask feature visualization. Column (a) shows the features without the soft-mask, (b) indicates features from the soft-mask images and (c) features after feature fusion from columns (a) and (b). Column (d) shows the feature fusion from the images without the applied soft-mask. The right side shows their corresponding templates with and without the soft-mask.

To get an effective and discriminative feature representation, we integrated the soft mask for both the template and search region in the proposed network. The proposed framework SCS-Siam takes four inputs including the template, soft mask template, search patch, and soft mask search patch, as illustrated in Figure 1. To obtain a better and efficient feature representation, we fused the features for soft masks at early convolutional layers. The soft mask features contain complementary localization information of the object, as shown in Figure 4. Feature fusion at early convolutional layers encodes the spatial target localization information and it increases the fidelity of the network to produce effective feature representation. The features for template and search branches are fused as:

(2) $$Z=B\left(T\right)+B\left(T_{sm}\right),$$

(3) $$X=B\left(S\right)+B\left(S_{sm}\right),$$

where B represents a convolutional block including a convolutional layer, a normalization layer, a rectifier layer, and a pooling layer. The proposed framework is defined as:

(4) g(T,S)=(Ω(Δ(ψ(Z)))⊕ψ(Z))∗ψ(X)+b,

where ψ(.) denotes the backbone network parameters, Δ(.) represents the proposed channel attention module, and Ω(.) shows the proposed spatial attentional module. The output of ψ(Z) is fed to the proposed channel attention as Δ(ψ(Z)). Then, this output is forwarded to the proposed spatial attention module as Ω(Δ(ψ(Z))). The output of spatial attentional module is element-wise added with ψ(Z). Then, finally, a response map is calculated from Equation (4). Please see Figure 1 for more details.

### 3.3. Channel Attention Module

We exploit the inter-channel relationship by integrating a soft channel-attentional mechanism into the proposed tracking framework. A special kind of visual pattern is captured by each deep channel during convolution. Deep channels behave differently and play a vital role to compute inference. Therefore, the channel attentional mechanism is a process of selecting important visual patterns for better inference. The channel attention module focuses on the most useful information for better discrimination. The objective of channel attention is to increase the adaption ability of the tracker by strengthening the most useful channels and reducing the impact of less important channels. SENet [69], SA-Siam [66], and RASNet [43] learn the channel attention using a max pooling and multi perception layer. In contrast to these, rather then employing single pooling layer, our channel exploits the channel relationships explicitly for both global max pooling and global average pooling using two separate sub-networks. The global average pooling exhibits the overall knowledge, whereas global average pooling indicates the finer object information for feature channels. The channels’ weight coefficients from our sub-networks yield better descriptor weights.

**Figure 5:**
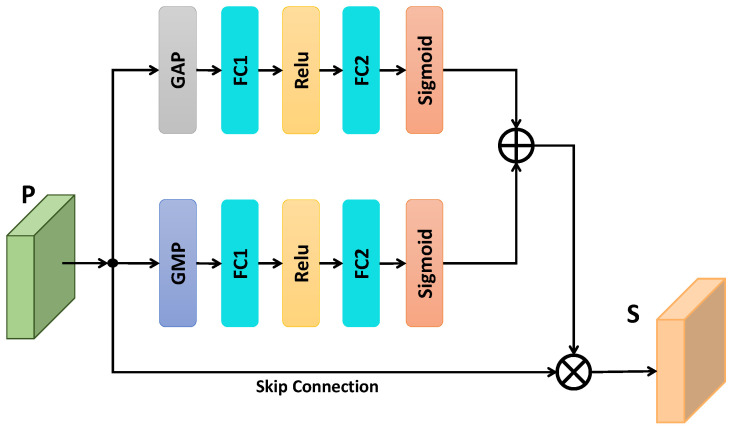
Proposed channel attentional mechanism. GAP means Global Average Pooling and GMP stands for Global Maximum Pooling. ⨁ indicates pixel-wise addition operation while ⨂ indicates the pixel-wise multiplication operation.

To exploit the channel attention, we proposed the light weight deep channel attentional network illustrated in Figure 5. Given an input feature map *P* containing *c* deep channels, features are forwarded to two independent sub-networks. Global Average Pooling (GAP) and Global Maximum Pooling (GMP) operations are performed for each sub-network separately to compute 1×1×c dimension descriptors. In each sub-network, the channels are decreased by a fully connected layer and then again increased using another fully connected layer. A Sigmoid activation is performed for each sub-network to compute descriptor weights. These descriptors are fused using an element-wise summation operation. The channel weights $\omega \in R^{c}$ are computed as:

(5) $$\begin{array}{cc} \omega =\mathrm{Sigmoid}(FC2(\mathrm{Relu}(FC1(GAP(P)))))\;\; & \\ & +\mathrm{Sigmoid}(FC2(\mathrm{Relu}(FC1(GMP(P))))). \end{array}$$

The weights ω are channel-wise multiplied with input features *P* to obtain *S* shown in Figure 5.

### 3.4. Spatial Attention Module

We also propose a spatial attention mechanism within our network to emphasize the important spatial locations of the target feature map. CBAM [37] computes spatial attention by computing global average and maximum pooling across channel to generate feature descriptor while DANet [40] exhibits more complexity to compute spatial attention. RASNet [43] utilizes a Guassian map to exploit spatial reliability. This approach has limitations, as an arbitrary object may contain contexual information and the network should give weights less to contextual information. On the other hand, the proposed spatial attention computes the global contextual descriptors using a simple model.

**Figure 6:**
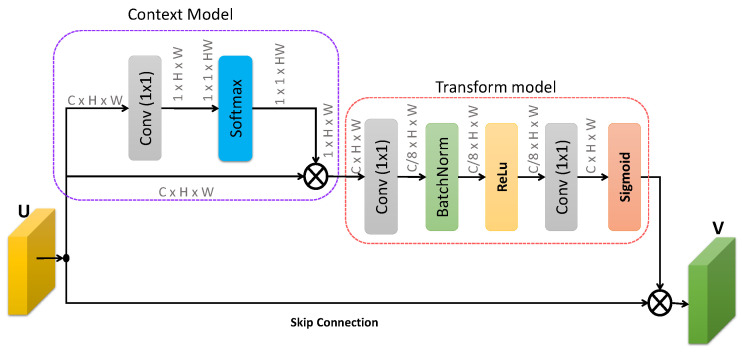
Proposed spatial attentional module. U represents the input feature map, V indicates the output feature map, and ⨂ indicates the element-wise multiplication operation. The softmax layer produces the same attention for all channels, while the later part after Sigmoid produces a different spatial attention across *C* channels.

In addition to the proposed soft-mask, the spatial attention mechanism highlights the important target locations within each channel in the latent space. Complementary to channel attention, our spatial attention module focuses on the most informative target regions within each channel. The output from the channel attention module is forwarded to the spatial attention module. The proposed spatial attention framework has two components including context and transform models, as shown in Figure 6. The context mode is responsible for computing the same spatial attention for all the feature channels. To compute the context model feature maps, we first apply a convolution layer to reduce the large number of channels to a single channel and then apply the softmax layer. The output from the softmax layer is multiplied with input feature maps, as shown in Figure 6. Contrary to the context model, transform is responsible for computing the different spatial attentions across the channels. To do so, the output of the context model is forwarded to multiple layers to first reduce the number of channels and then increase the number of channels. Then a sigmoid activation function is applied and finally the output is achieved using a skip connection from the input feature maps.

### 3.5. Network Training

The proposed network is trained as a generalized object tracker by learning on a large benchmark GOT-10K dataset [70], which is composed of 1.5 million annotated frames and more than 10000 video sequences. Similar to SiameseFC [30], we define a template patch size of 127×127×3 and a search region size of 255 × 255 × 3. We also generate a template with a soft mask of size 127×127×3 and a search with a soft mask of size 255 × 255 × 3. The network is trained offline using Stochastic Gradient Descent (SGD) to yield a response map $g(T_{k},T_{k}^{sm},S_{k},S_{k}^{m})$ for input images and a $Y_{k}$ ground truth map, where $T_{k}$ represents the template image, $T_{k}^{sm}$ shows the template with soft mask, $S_{k}$ denotes the search, and $S_{k}^{sm}$ refers to the search with a soft mask, as shown in Figure 1. The network parameters θ were trained to minimize the following loss function:

(6) $$\underset{\theta }{\mathrm{argmin}}\frac{1}{K}\sum_{k=1}^{K}L\left(g\left(T_{k},T_{k}^{sm},S_{k},S_{k}^{sm}\right),Y_{k}\right),$$

where θ represents the network parameters, *K* represents the total number of training samples, and L(.) represents the logistic loss function computed as:

(7) $$L\left(g_{k},y_{k}\right)=\frac{1}{\left|\delta \right|}\sum_{(i,j)\in \delta }log(1+exp\left(-g_{k}\left(i,j\right).y_{k}\left(i,j\right)\right),$$

where $g_{k}\left(i,j\right)$ and $y_{k}\left(i,j\right)\in \left\{+1,-1\right\}$ represent the similarity value and ground truth label corresponding to the (i,j)th position on the score map, δ represents the set of positions in the search window on the score map.

We present the training and testing algorithm for our proposed framework in Algorithm 1 and Algorithm 2, respectively. 

**Algorithm 1:** Offline Training of the proposed framework

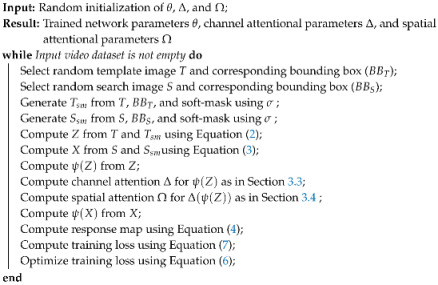



**Algorithm 2:** Tracking of the proposed method

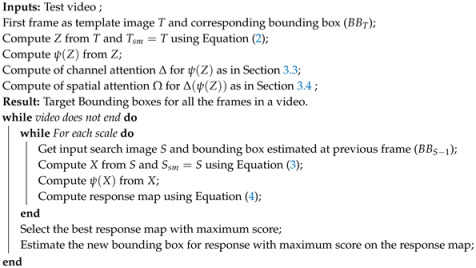



## 4. Experiments and Results

We propose three different versions of tracking framework including Soft mask with Channel and Spatial attention Siamese (SCS-Siam), Soft mask with Channel Attention Siamese (SCA-Siam), and Soft mask with Spatial Attention Siamese (SSA-Siam). We evaluate the proposed tracking versions on five benchmark datasets including OTB2015 [44], TempleColor128 [45], UAV123 [46], VOT2016 [47], and VOT2017 [48]. We compare its performance with 39 state-of-the-art methods including TRACA [71], SRDCF [72], UDT [73], SiamtTri [31], SiameseFC [30], Staple [74], CFNet [34], CNNSI [75], RNN [76], MLT [77], P2FNet [78], SiamFc-lu [79], DSiamM [57], Li et al. [80], Kuai et al. [81], SINT [82], HASiam [83], ECO [21], MEEM [84], SAMF [85], MUSTER [86], CSK [6], CMKCF [87], SATIN [88], ACT [89], MemTrack [58], DSiam [90], GradNet [91], SiameseRPN [92], ACFN [93], CSRDCF [68], SCT [94], KCF [7], SSKCF [47], DPT [95], DSST [96], CCOT [20], SiamDCF [97], and UCT [98].

### 4.1. Implementation Details

The proposed tracker was trained offline to attain generalization on GOT-10K [70]. The model was trained using the Stochastic Gradient Descent (SGD) method to minimize Equation (6). The network parameters are shown in Table 1. We set the weight decay to $10^{-5}$ and momentum to 0.9. The initial learning rate was set to $10^{-2}$ and decreased exponentially until reaching $10^{-5}$. During training, the background information weight in the generation of the soft mask in Section 3.2 was set to 0.9, and the target bounding box information was set to 1. To improve the tracking performance, we used larger images during inference. The sizes for the input images of both the exemplar and search branches were set to 135×135×3 and 263×263×3 respectively. During testing, we did not compute the soft mask but instead cloned the template and search region to compute inference. To address the scale variations across consecutive video frames, we constructed a pyramid for the target consisting of three different scales $\left\{1.0375^{-1},1,1.0375\right\}$ at the current frame based on the previous target position. During inference, we got three response maps and we selected the best response matching the target scale. Code was implemented in Python 3.7 using PyTorch. Experiments were performed on a machine having Intel i7 3.6 GHz processor, 32 GB of RAM, and an NVIDIA TITAN XP GPU card. The average tracking speed of the proposed tracker was 73 frames per second (FPS).

### 4.2. Datasets and Evaluation Metrics

OTB2015 [44] contains 100 fully annotated sequences with 11 different tracking challenges. Temple Color-128 (TC128) [45] is also a widely used tracking benchmark. Compared to OTB2015, it has 128 challenging videos and also contains 11 tracking challenges. The UAV123 dataset consists of 123 videos captured from Unmanned Aerial Vehicle (UAV) at a low-altitude and all videos are fully annotated [46]. We used One Pass Evaluation (OPE) for evaluation. Performance evaluations are performed using precision and success to measure for the aforementioned datasets. The former metric is computed as the Euclidean distance between the center location of the ground truth and the predicted location and is defined as the percentage of the frames where the Euclidean distance lies within a 20 pixels threshold. The latter metric computes the Overlap Score (OS) using an intersection over union. A frame is considered a success if its OS exceeds a threshold of 0.50. The comparisons were conducted over the VOT2016 and VOT2017 datasets. During the evaluation of these datasets, the tracker was re-initialized if it failed. We used the official toolkit and three parameters including the Expected Average Overlap (EAO), Robustness (R), and accuracy (A) to compare the tracking performance. Details of the aforementioned datasets are presented in Table 2.

### 4.3. Experiments on OTB2015

**Figure 7:**
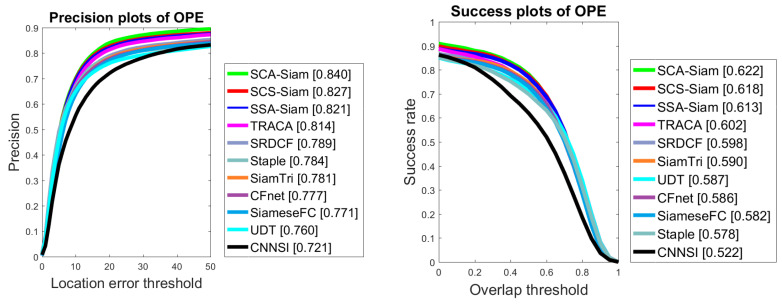
Comparison over OTB2015 in terms of precision and success.

On OTB2015 dataset, we compared our algorithms with eight existing state-of-the-art algorithms including TRACA [71], SRDCF [72], UDT [73], SiamtTri [31], SiameseFC [30], Staple [74], CFNet [34], and CNNSI [75]. Our proposed SCA-Siam, SCS-Siam and SSA-Siam trackers demonstrated better performance compared to TRACA and all other trackers in terms of both precision and success over OTB2015, as illustrated in Figure 7. SCA-Siam gained 62.2% success, which is 2% higher than TRACA. Likewise, SCA-Siam algorithm obtained 2.6% more precision than TRACA over OTB2015.

We also compared our SCA-Siam, SSA-Siam, and SCS-Siams version with other various state-of-the-art trackers over OTB2015. Table 3 presents the comparison of various trackers based on three parameters precision, success, and speed in FPS. The performance of our algorithm SCA-Siam surpassed the other methods in terms of precision and success. TRACA performed tracking at 101 FPS, but its precision and success score were less than our SCA-Siam by 2.6% and 2% respectively. Kuai et al. [81] secured 62.2% success but exhibited less precision and speed than our algorithms.

**Figure 8:**
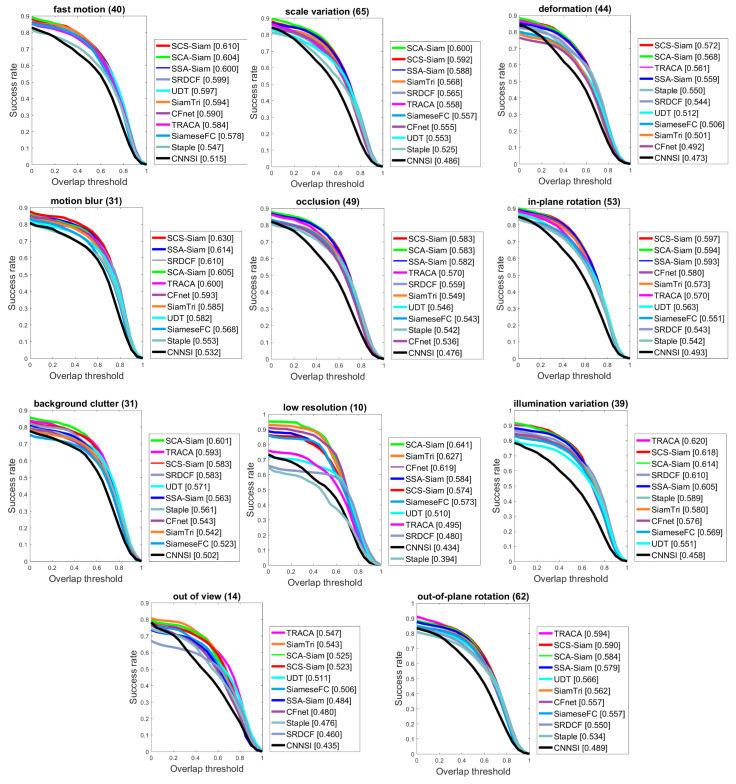
Comparison for eleven different challenges in terms of success over OTB2015.

We also conducted the experiments to exploit the robustness for the SCA-Siam, SSA-Siam, and SCS-Siam algorithms for 11 tracking challenges including occlusion, deformation, fast motion, motion blur, light and scale variations, and others. We selected the OTB2015 benchmark to evaluate for different challenges, as presented in Figure 8 and Table 4. We observed that the proposed algorithm SCS-Siam secured first rank in terms of success when compared with other trackers for five challenges: fast motion, occlusion, deformation, motion blur, and in-plane rotation, as shown in Figure 8. Our SCA-Siam showed better performance for three challenges such as scale variations, background clutter, and low resolution in terms of success. However, our SCS-Siam revealed slightly less performance than TRACA for two groups of videos including illumination variations and out-of-plane rotation. Table 4 presents the attribute-based performance in terms of precision scores for eight state-of-the-art trackers. Our SCA-Siam demonstrated best performance for nine challenges including Fast Motion (FM), Background Clutter (BC), Deformation (Def), Illumination Variations (IV), Occlusion (OCC), In-Plane Rotation (IPR), Low Resolution (LR), Out of View (OV), and Scale Variations (SV). Our SCS-Siam showed better performance for the Motion Blur (MB) challenge in terms of precision. TRACA showed best performance for Out-of-Plane Rotation (OPR), while our SCA-Siam ranked second.

**Figure 9:**
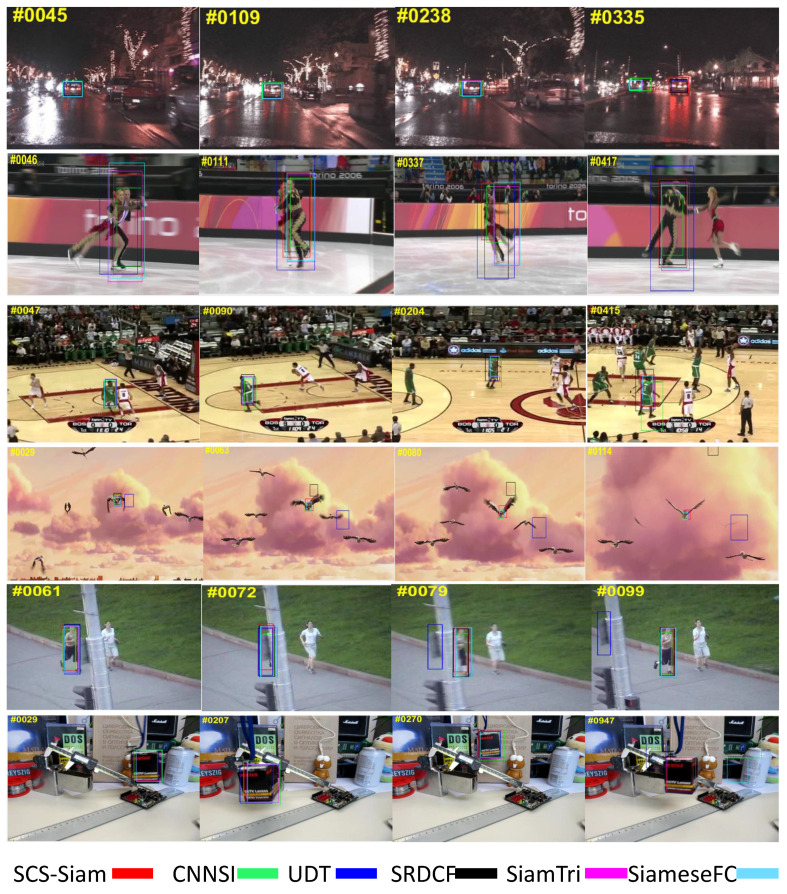
Qualitative study of our method with state-of-the-art trackers over sequences including *CarDark*, *Skating*2-2, *Basketball*, *Bird*2, *Jogging*-1, and *Box*.

We further compared the tracking results qualitatively with CNNSI, UDT, SRDCF, SiamTri, and SiameseFC trackers, as illustrated in Figure 9. We preformed qualitative experiments for selected sequences from OTB2015 such as *CarDark*, *Skating*2-2, *Basketball*, *Bird*2, *Jogging*-1, and *Box* videos. Our SCS-Siam tracker did not lose the target object for any sequence, as illustrated in Figure 9.

### 4.4. Experiments on TC128 and UAV123

We evaluated the performance for TC128 and UAV123 datasets using precision and success. Table 5 presents the comparison of the proposed method over TC128 with the following trackers: SCT [94], KCF [7], CNNSI [75], ACT [89], UDT [73], CFNet [34] and baseline tracker SiameseFC [30]. Our algorithm SCS-Siam showed better performance compared to other trackers in terms of precision and achieved the highest scores 74.2%. While our SSA-Siam secured best overlap 54.2%. KCF is computationally effective, exhibiting its tracking 160 FPS; however, our SCA-Siam, SSA-Siam, and SCS-Siam surpassed the KCF for both success and precision.

**Figure 10:**
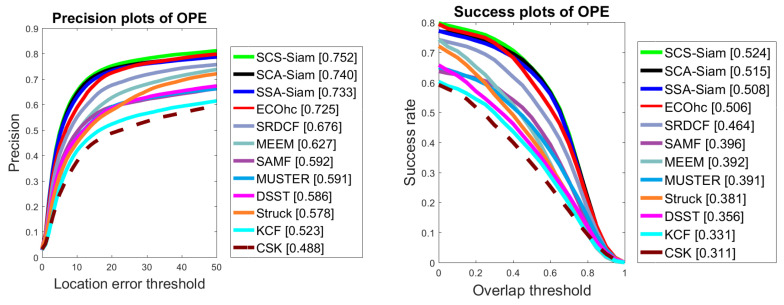
Comparison over UAV123 in terms of precision and success.

We validate the performance of our trackers over the UAV123 dataset using precision and success. Figure 10 demonstrates the plots for precision and success for the UAV123 dataset. We note that SCS-Siam surpassed other methods in performance and achieved the best scores for precision (75.2 %) and overlap (52.4%).

### 4.5. Experiments on VOT2016 and VOT2017

Table 6 presents the Expected Average Overlap (EAO), accuracy (A), and Robustness (R) of the compared trackers over VOT2016 [47] for the baseline experiments. Our methods are compared with 11 trackers: MemTrack [58], MemDTC [90], ECO [21], SRDCF [72], DSiam [57], CMKCF [87], Staple [74], CCOT [20], UDT [73], and SiameseFC [30]. CCOT obtained the maximum EAO score, and its robustness value is larger than our proposed algorithm, and overlap scores is less than our algorithm, as presented in Table 6. Overall, our algorithms demonstrated the better accuracy and robustness. Moreover, our algorithms showed the highest accuracy score against all the compared trackers.

We compute Expected Average Overlap (EAO), accuracy (A), and Robustness (R) to perform comparison over VOT2017 [48] in Table 7. We compared our algorithms with 12 other trackers: CSRDCF [68], MemTrack [58], MemDTC [90], SRDCF [72], DSST [96], SATIN [88], SiamDCF [97], UCT [98], SiameseFC [30], GradNet [91], and SiameseRPN [92]. CSRDCF surpassed our method for both EAO (0.25). However, our trackers achieved more accuracy, low robustness, and demonstrated superior computational efficiency compared to CSRDCF. SATIN has a high EAO of 0.28, but our algorithms have comparatively high overlap scores and low robustness. Furthermore, our SCS-Siam obtained a favorably high overlapping score and low robustness compared to the other trackers.

### 4.6. Ablation Study

We performed an extensive ablation study to validate the effectiveness of different components of the proposed method. We performed experiments for each version and compared the results using precision and success over OTB2015 [44]. We also compared our methods with the baseline SiameseFC tracker, and evaluated the effectiveness of the three integrated modules.

First, we performed a series of experiments to select the best σ for soft mask generation. During training, we set different values of σ to weight background information to generate soft-mask images. During testing, we set the background weight to 1 for all experiments and present the performance in Table 8 in terms of precision and success. For example, in Table 8, SCS-Siam-0.0 means that SCS-Siam was trained with σ=0.0 such that it has no background information to generate soft mask images. Similarly, SCS-Siam-0.3σ=0.3 was used during training. We observe that SCS-Siam-0.9 exhibited the best performance where soft-mask images were generated when σ was set with σ=0.9. In an additional experiment, we also tested the proposed SCS-Siam with soft-masks generated with σ=0.9 during the test time as well. We observed that this choice showed degradation in performance compared to σ=1. It is because that the target estimation at previous frame may not be accurate due to distractors. Therefore, this degradation in performance may be due to the construction of an inaccurate soft mask from the previous frame.

In Table 9, we present the comparison of different versions of the proposed framework. SiameseFC is a baseline tracker trained over the ImageNet-ILSVRC2015 dataset and the SiameseFC* is trained over GOT-10k dataset. The proposed framework is referred to as the ‘Extended-SiameseFC’, which is trained over GOT-10K without the soft-mask. We notice that SiameseFC* performed better than the SiameseFC, which is because the earlier was trained over a larger dataset compared to the former. We also observe that the proposed Extended-SiameseFC showed improved tracking performance than SiameseFC*. Because of the low level feature fusion, it achieved better localization. We compared our three versions of the proposed algorithm including SCA-Siam, SSA-Siam, and SCS-Siam to validate the integration of channel attention module, spatial attention module, and both modules, respectively. SCA-Siam refers to the channel attention module integrated in the proposed framework, while SSA-Siam represents the proposed spatial attention. SCS-Siam indicates that the proposed framework contains both channel and spatial attention modules. Table 9 shows that SCA-Siam achieved better performance compared to SCS-Siam and SSA-Siam trackers. We also investigate the order of the spatial and channel attention in the proposed framework. SCS-Siam means that it has a channel attention followed by the spatial attention module. While SSC-Siam represents the tracker that has spatial attention followed by channel attention. The experimental results demonstrate that the inclusion of the channel attention module before the spatial attention shows superior tracking performance. We also integrate the channel and spatial attention modules for exemplar and search branches within the proposed framework denoted as B-SCS-Siam in Table 9. It can be seen that no performance gain is obtained, whereas the speed decreases when channel and spatial attentions are applied for both exemplar and search branches.

Proposed channel attention and spatial attention modules consist of two sub-networks, as shown in Figure 5 and Figure 6, respectively. We performed different experiments to validate the importance of each sub-network for both channel and spatial attention modules, as shown in Table 10. For SCA-Siam, we performed experiments for each sub-network. For example, SCA-GMP indicates the SCA-Siam that utilizes global maximum pooling sub-network only while SCA-GAP represents the SCA-Siam that has a global average pooling sub-network only. Similar to SCA-Siam, we also performed two experiments for SSA-Siam. SSA-Context represents the SSA-Siam without transform sub-network while SSA-Transform indicates the SSA-Siam without context sub-network. Experimental results in Table 10 revealed to us that each sub-network of the channel and spatial attention modules is important to achieve better tracking performance.

## 5. Conclusions

In this work, a soft mask based spatial feature fusion method is proposed which is further strengthened with channel and spatial attention mechanisms. The proposed approach is embedded within a Siamese framework to obtain improved tracking accuracy and robustness. The soft mask based feature fusion mechanism produces efficient and discriminative features for effective tracking. During training, soft-masks were used to highlight the target information region. Features from original and soft-mask images were fused for both template and search branches, which enhanced the tracker’s localization ability. The channel attention mechanism was utilized to exploit the rich feature channels for discrimination while reducing the weights for less informative channels. A spatial attention module was also proposed to enhance the inter-channel localization ability of the tracker. The proposed framework was evaluated over five publicly available tracking benchmark datasets exhibiting significant improvement over 39 state-of-the-art trackers.

## Figures and Tables

**Table 1:** Network parameters θ of the proposed framework. Features are fused after the Max-Pool1 layer for both exemplar and search branches, as shown in Figure 1. The network Ψ(·) in Figure 1 contains Conv2, Conv3, Max-Pool2, Conv4, Conv5, and Conv6 layers.

Layer	Filter Size	Stride	In and Out Channel	Template and Template with Soft-Mask	Search and Search with Soft-Mask
Input			3	3×135×135	3×263×263
Conv1	11×11	2	3×192	192×63×63	192×127×127
Max-Pool1	3×3	2	-	192×31×31	192×63×63
Feature Fusion	-	-	-	192×31×31	192×63×63
Conv2	3×3	1	192×256	256×29×29	256×61×61
Conv3	3×3	1	256×256	256×27×27	256×59×59
Max-Pool2	3×3	2	-	256×13×13	256×29×29
Conv4	3×3	1	256×512	512×11×11	512×27×27
Conv5	3×3	1	512×512	512×9×9	512×25×25
Conv6	3×3	1	512×384	384×7×7	384×23×23

**Table 2:** Details of different benchmarks.

Benchmarks	OTB2015	TC-128	UAV123	VOT2016	VOT2017
Sequences	100	128	123	60	60
Minimum frames	71	71	109	48	41
Mean frames	590	429	915	357	356
Max frames	3872	3872	3085	1500	1500
Total frames	59,040	55,346	1,12,578	21,455	21,356

**Table 3:** Comparison of the proposed algorithm with various state-of-the-art methods over OTB2015 using precision, success, and speed in Frames Per Second (FPS). Bold values represent the maximum value for precision, success and FPS.

Trackers	Precision	Success	FPS	Real-Time
RNN [76]	79.8	60.6	-	-
MLT [77]	-	61.1	48	Yes
P2FNet [78]	79.8	59.5	52	Yes
SiamFc-lu [79]	-	62.0	82	Yes
DSiamM [57]	-	60.5	18	No
SiamTri [31]	78.1	59.0	85	Yes
Li’s [80]	77.1	58.2	30	Yes
CNNSI [75]	72.1	52.2	1<	No
Kuai et al. [81]	82.2	**62.2**	25	No
TRACA [71]	81.4	60.2	**101**	Yes
SINT [82]	-	59.2	4	No
ACFN [93]	80.2	57.5	15	No
CSRDCF [68]	73.3	58.7	13	No
Staple [74]	78.4	57.8	80	Yes
SRDCF [72]	78.9	59.8	6	No
HASiam [83]	81.0	61.1	30	Yes
UDT [73]	76.0	59.4	70	Yes
SiameseFC-G&M [99]	81.0	61.3	68	Yes
CMKCF [87]	82.2	61.0	74.4	Yes
CFNet [34]	77.7	58.6	43	Yes
SiameseFC [30]	77.1	58.2	86	Yes
SCA-Siam (Ours)	**84.0**	**62.2**	76	Yes
SSA-Siam (Ours)	82.1	61.3	75	Yes
SCS-Siam (Ours)	82.7	61.8	73	Yes

**Table 4:** Comparison for eleven different challenges in terms of precision over OTB2015. Bold values represent the maximum value for precision.

Trackers	SiamTri	CFNet	SRDCF	TRACA	SiameseFC	UDT	Staple	CNNSI	SCA-Siam	SCA-Siam	SCS-Siam
**FM**	77.6	77.4	77.3	76.2	75.8	75.3	72.9	67.5	**80.2**	78.3	79.2
**BC**	71.5	73.1	77.5	79.9	69.0	74.9	74.9	68.7	**80.2**	74.6	77.5
**MB**	74.4	76.1	78.2	77.1	72.4	71.4	71.9	69.6	78.2	77.9	**79.2**
**Def**	68.0	66.9	73.4	76.9	69.0	67.0	75.1	68.7	**80.0**	77.6	79.3
**IV**	75.1	76.3	78.7	83.3	74.0	70.0	78.2	60.0	**83.6**	81.2	83.3
**IPR**	75.9	78.5	73.7	79.4	72.8	74.1	75.1	68.8	**81.1**	80.5	80.5
**LR**	88.4	86.1	63.1	73.1	81.5	68.8	59.1	66.0	**91.9**	83.0	82.5
**OCC**	72.6	71.3	73.5	77.5	72.2	70.6	72.8	64.4	**78.5**	77.1	77.3
**OPR**	76.1	75.8	74.4	**82.8**	75.4	74.7	73.7	68.9	80.6	79.8	80.3
**OV**	72.3	65.0	59.7	70.0	66.9	65.1	66.8	59.4	**73.0**	65.6	70.3
**SV**	75.2	74.8	74.9	76.9	73.9	71.4	73.1	68.7	**81.6**	79.2	79.6

**Table 5:** Comparison of the proposed method with various state-of-the-art methods over TC128 using precision, success, and speed in FPS. Bold value represent the maximum value for precision, success and FPS.

Trackers	Precision	Success	FPS
SCT [94]	62.7	46.6	40
KCF [7]	54.9	38.7	**160**
CNNSI [75]	63.8	45.6	<1
UDT [73]	65.8	50.7	70
CFNet [34]	60.7	45.6	43
ACT [89]	73.8	53.2	30
SiameseFC [30]	68.8	50.3	86
SCA-Siam (Ours)	73.1	53.2	76
SSA-Siam (Ours)	73.8	**54.2**	75
SCS-Siam (Ours)	**74.2**	53.8	73

**Table 6:** Performance comparison for different trackers over VOT2016. Bold values represent the maximum value for overlap, robustness, and EAO.

Trackers	Overlap (↑)	Robustness (↓)	EAO (↑)
MemTrack [58]	0.53	1.44	0.27
MemDTC [90]	0.51	1.82	0.27
ECO [21]	0.54	-	0.37
Staple [74]	0.53	0.38	0.29
SRDCF [72]	0.54	0.42	0.25
DSiam [57]	0.49	2.93	0.18
CCOT [20]	0.54	0.24	**0.33**
UDT [73]	0.54	-	0.22
SiameseFC [30]	0.53	0.46	0.23
CMKCF [87]	0.53	**0.18**	0.30
SCA-Siam (Ours)	**0.55**	0.23	0.28
SSA-Siam (Ours)	**0.55**	0.23	0.27
SCS-Siam (Ours)	**0.55**	0.21	0.28

**Table 7:** Performance comparison for different trackers over VOT2017. Bold values represent the maximum value for overlap, robustness, EAO, and FPS.

Trackers	Overlap (↑)	Robustness (↓)	EAO (↑)	FPS
CSRDCF [68]	0.49	0.49	0.25	13
MemTrack [58]	0.49	1.77	0.24	50
MemDTC [90]	0.49	1.77	0.25	40
SRDCF [72]	0.49	0.97	0.12	6
DSST [96]	0.39	1.45	0.08	24
SATIN [88]	0.49	1.34	**0.28**	24
SiamDCF [97]	0.50	0.47	0.25	60
UCT [98]	0.49	0.48	0.20	41
SiameseFC [30]	0.50	0.59	0.19	86
GradNet [91]	0.50	0.37	0.24	80
SiameseRPN [92]	0.49	0.46	0.24	200
SCA-Siam (Ours)	0.51	0.36	0.21	76
SSA-Siam (Ours)	**0.52**	0.38	0.20	75
SCS-Siam (Ours)	**0.52**	**0.29**	0.24	73

**Table 8:** Performance of the proposed SCS-Siam over the OTB2015 dataset using different values of σ to generate soft mask images during training as shown (see Section 3.2). ¶ denotes that σ is set at 0.9 during training as well as testing. However, σ is set at 1 for the test time for the rest of the experiments.

SCS-Siam-σ	Precision	Success
SCS-Siam-0.0	76.3	56.5
SCS-Siam-0.3	78.2	58.6
SCS-Siam-0.5	81.5	60.4
SCS-Siam-0.7	80.0	59.6
SCS-Siam-0.9	82.7	61.8
SCS-Siam-1.0	79.7	59.5
SCS-Siam-$0.9{\;}^{¶}$	81.7	60.5

**Table 9:** Comparison of different versions of the proposed framework on the OTB2015 dataset. SiameseFC is the baseline network, SiameseFC* shows that SiameseFC was retrained on GOT-10k dataset, Extended-SiamFC means the proposed framework without a soft mask and attentions, SSA-Siam is Extended-SiamFC with a soft mask and spatial attention, SCA-Siam is Extended-SiamFC with a soft mask and channel attention, SSC-Siam is Extended-SiamFC with a soft mask and spatial attention followed by channel attention, and SCS-Siam is the proposed framework with soft mask, channel attention followed by spatial attention. B-SCS-Siam means that the channel attention was followed by spatial attention at both exemplar and search branches.

Tracker	Precision	Success	FPS
SiameseFC	77.1	58.2	86
SiameseFC*	79.2	59.7	86
Extended-SiamFC	80.3	60.2	79
SSA-Siam	82.1	61.3	75
SCA-Siam	84.0	62.2	76
SSC-Siam	82.0	60.9	73
SCS-Siam	82.7	61.8	73
B-SCS-Siam	81.5	60.4	59

**Table 10:** Ablation study of channel and spatial attention modules. SCA-GMP is the SCA-Siam using only global max pooling sub-network while SCA-GAP represents the SCA-Siam with only the global average pooling sub-network. Similarly, SSA-Context means the SSA-Siam with the context modeling sub-network, while SSA-Transform represents the SSA-Siam with the transform sub-network alone.

Tracker	Precision	Success
SCA-GMP	83.5	61.8
SCA-GAP	83.9	62.0
SCA-Siam	84.0	62.2
SSA-Context	81.5	60.8
SSA-Transform	81.8	61.0
SSA-Siam	82.1	61.3
